# Feasibility, Safety and Preliminary Efficacy of 1:1 THC:CBD Cannabis Oil for Fibromyalgia Symptoms: Results From a Randomised, Double‐Blind, Placebo‐Controlled Pilot Trial

**DOI:** 10.1155/prm/7311235

**Published:** 2026-05-16

**Authors:** Inna Kurlyandchik, Evelin Tiralongo, Romy Lauche, Gerald Tracey, Jennifer Ng, Janet Schloss

**Affiliations:** ^1^ National Centre for Naturopathic Medicine, Southern Cross University, Lismore, New South Wales, Australia, scu.edu.au; ^2^ School of Pharmacy and Medical Sciences, Griffith University, Gold Coast, Queensland, Australia, health.qld.gov.au; ^3^ Gold Coast University Hospital, Southport, Queensland, Australia, health.qld.gov.au; ^4^ Pacific Rheumatology, Southport, Queensland, Australia, health.qld.gov.au; ^5^ Griffith University-Gold Coast Campus, Southport, Queensland, Australia, health.qld.gov.au

## Abstract

Fibromyalgia is a chronic disorder characterised by widespread pain and other symptoms that substantially impact the quality of life. This double‐blind, randomised, placebo‐controlled trial primarily assessed feasibility (procedures and intervention adherence) and safety/tolerability of a 1:1 delta‐9‐tetrahydrocannabinol:cannabidiol (THC:CBD) cannabis oil (10 mg/mL each) in 24 adults with fibromyalgia, with secondary, preliminary assessment of efficacy across symptom domains. Participants completed a 4‐week dose titration followed by 12 weeks of stable dosing. Of 77 prescreened individuals, 24 were randomised, yielding a screening‐to‐enrolment ratio of approximately 3:1 (31.2%). Recruitment reached 66.7% of the target (24/36); the shortfall was mainly due to geographic and legal barriers. Retention was 91.7% (22/24) and adherence was high, with all participants taking ≥ 90% of the prescribed doses. The study medication was well tolerated in this small sample, with adverse events mostly mild and no serious events observed. Secondary outcomes suggested medium to large between‐group effects favouring cannabis for pain reduction, improved sleep quality, and reduced fibromyalgia impact (FIQR), but findings should be interpreted cautiously given the small sample. Clinically meaningful FIQR improvement (predefined MCID 45.5%) occurred in 40% of the cannabis‐treated participants versus 10% with placebo. For pain, 70% of the cannabis group reported ≥ 30% reduction post‐titration and at Week 12 (Placebo 20% and 40%, respectively). Fatigue and anxiety/depression showed no significant changes. A randomised trial of 1:1 THC:CBD oil appears feasible with excellent retention and adherence, though recruitment barriers need addressing. Preliminary safety and efficacy signals warrant confirmation in larger, adequately powered trials.

**Trial Registration:** Australian New Zealand Clinical Trials Registry: ACTRN12623000345684

## 1. Introduction

Fibromyalgia is a chronic widespread pain syndrome affecting an estimated 3%–5% of the population, disproportionately women [1]. Characterised by musculoskeletal pain, fatigue, sleep disturbance, cognitive dysfunction and psychological distress, fibromyalgia significantly impacts patients’ quality of life and functional ability [2]. The pathophysiology is not fully understood but involves the sensitisation of neural pain pathways in the central nervous system, with possible contributions from the peripheral nervous system [3]. While considered a spectrum disorder with variable symptom presentation and severity, fibromyalgia often co‐occurs with other functional somatic syndromes and mental health conditions such as irritable bowel syndrome, chronic fatigue syndrome and mood and anxiety disorders [2].

Treatment strategies for fibromyalgia combine nonpharmacological and pharmacological approaches [2]. Nonpharmacological interventions, such as tailored physical therapy and psychoeducational programs, are considered primary treatment modalities and tend to achieve better outcomes than pharmacological interventions alone [2]. Pharmacological approaches play a supportive role in symptom management, targeting pain, sleep disturbance and psychological distress [2]. Evidence supports the use of certain medications, including amitriptyline, duloxetine, milnacipran and pregabalin, while opioids are generally avoided due to poor clinical response and risk of hyperalgesia [2, 4]. However, the efficacy of these drugs is often limited, and many patients either experience side effects or do not benefit from them [2]. Furthermore, the high cost of prescription medicines often leads to reliance on potentially ineffective over‐the‐counter medications like paracetamol and nonsteroidal anti‐inflammatory drugs [5].

In recent years, medicinal cannabis has emerged as a potential therapeutic option for fibromyalgia, with increasing patient interest in new treatment approaches [5]. Given fibromyalgia’s complexity and diverse manifestations, investigating multifaceted therapies such as medicinal cannabis is well justified [2].

*Cannabis sativa* L. is a plant with a long history of medicinal use, containing over 100 phytocannabinoids, including delta‐9‐tetrahydrocannabinol (THC) and cannabidiol (CBD) [6]. These compounds interact with the endocannabinoid system, which plays a critical role in modulating pain, mood and sleep—key domains affected in fibromyalgia [7–9]. Consequently, cannabis is being investigated as a potential therapeutic agent for fibromyalgia, with several studies suggesting it may help reduce pain, improve sleep and enhance the quality of life, although findings to date remain mixed and optimal dosing strategies, including the THC:CBD ratio and routes of administration, still require further investigation [10].

As research progresses, feasibility studies are critical for assessing the practicality, safety and preliminary efficacy of medicinal cannabis interventions before larger clinical trials are conducted [11]. This trial evaluates the feasibility and safety of an oral cannabis oil formulation with a 1:1 THC:CBD ratio (10 mg/mL each), alongside preliminary assessments of its efficacy for fibromyalgia symptoms.

## 2. Methods

### 2.1. Trial Design and Setting

This was a single‐centre, double‐blind, randomised placebo‐controlled feasibility trial conducted at the Clinical Trial Unit (CTU) of Griffith University on the Gold Coast, Australia. The trial was approved by the Human Research Ethics Committees of Southern Cross University (approval number 2022/146) and Griffith University (approval number 2023/137). The trial was conducted in accordance with the Declaration of Helsinki [12], the International Council for Harmonisation of Technical Requirements for Pharmaceuticals for Human Use (ICH) Good Clinical Practice (GCP) guidelines [13] and the National Statement on Ethical Conduct in Human Research [14].

This trial is reported in accordance with the CONSORT 2010 guidelines and extension to randomised pilot and feasibility trials [15].

### 2.2. Sample Size

A sample size of 30 participants is considered adequate for a feasibility trial based on Browne’s flat rule of thumb for two‐armed pilot trials [16]. This trial aimed to recruit 36 participants in total (18 per study arm), allowing for an estimated 20% attrition. The sample size was intended to provide sufficient data to assess recruitment, retention, adherence and safety parameters [15].

### 2.3. Participants

Participants were required to be at least 18 years old, able to provide written informed consent and capable of completing outcome assessments. Candidates were required to have a confirmed diagnosis of fibromyalgia, which meets the 2016 American College of Rheumatology criteria [17]. The diagnosis was verified at screening by the Principal Clinical Investigator or Clinical Subinvestigator (both rheumatology clinicians). Participants also had to have an Average Daily Pain Score (ADPS) ≥ 4 on an 11‐point numeric rating scale over the 7‐day period before randomisation [18]. Women of childbearing potential were expected to use highly effective contraception during the study and for 4 weeks poststudy.

Exclusion criteria comprised clinically significant unstable medical conditions, significant laboratory abnormalities, pain from other confounding conditions (e.g., diabetic neuropathy or widespread inflammatory musculoskeletal disorders) and recent malignancies (other than benign skin cancers). Individuals with moderate severe to severe depression or anxiety, high suicide risk, any lifetime psychotic or bipolar disorder or current substance abuse or dependence were also excluded. Additional criteria were hypersensitivity to cannabis products, tree nut allergy, pregnancy or breastfeeding. Exclusion also applied to those using antipsychotics (except low‐dose ones for sleep), chemotherapy, immunosuppressive therapies or warfarin. Participants were also excluded if currently using cannabis or medicinal cannabis product (verified by urinary THC testing at screening) and unable to undergo a 30‐day washout. A full list of inclusion and exclusion criteria is provided in Supporting Information SI1.

### 2.4. Recruitment

Participants were recruited over approximately six months (August 2023 to January 2024) via referrals from primary investigators and the Rheumatology Department at Gold Coast University Hospital and social media campaigns targeting fibromyalgia Facebook groups in Brisbane, Gold Coast and across Australia. The study utilised a rolling recruitment, which included online and telephone prescreening and an in‐person eligibility assessment. All participants provided informed written consent prior to enrolment.

### 2.5. Randomisation and Blinding

Eligible participants were randomly assigned in a 1:1 ratio to receive either medicinal cannabis oil or placebo. The randomisation sequence was computer generated using sealed-envelope.com based on block randomisation with varying block lengths ranging from 2 to 6; no stratification was applied. The sequence was provided directly to unblinded pharmacy personnel, who did not participate in participant recruitment or management, data collection or analysis. Allocation concealment was maintained through opaque labelling. These personnel sequentially assigned and labelled the study medication and placebo containers with participant identification numbers according to the randomisation sequence before dispensing, ensuring blinding was preserved for all other study personnel. Participants, investigators, nurses and outcome assessors remained blinded throughout the trial.

Both formulations were dispensed in identical amber glass bottles with opaque, participant‐coded labels. The placebo was formulated with MCT, walnut and avocado oils to approximate the colour, texture and smell of the active oil; however, taste was not specifically matched. The parallel design meant participants could not directly compare formulations.

### 2.6. Intervention: Study Drug, Titration and Dosing

The study medication was cannabis oil containing equal concentrations of delta‐9‐THC and CBD at 10 mg/mL each, administered orally. This product is investigational and has not been approved by the US Food and Drug Administration (FDA) or the Australian Therapeutic Goods Administration (TGA) for fibromyalgia. The 1:1 THC:CBD ratio was selected following consultations with practising Australian medicinal cannabis physicians to identify commonly used ratios for fibromyalgia, aiming to reflect real‐world clinical practice. The placebo was an oil‐based product, with small amounts of walnut and avocado oil added to closely match the colour, texture and smell of the study medication. Both formulations used medium‐chain triglyceride (MCT) oil as the carrier.

Participants were instructed to take the oil at night, approximately 1 hour before bedtime. For titration, all participants started with a dose of 0.25 mL for 2 days. Once‐daily evening dosing was chosen to prioritise adherence and operational simplicity in this feasibility trial, target nocturnal symptoms (sleep disturbance) and minimise daytime psychoactive and other adverse effects (AEs) associated with higher or divided THC doses, consistent with pragmatic dosing guidance for oral cannabinoids [19]. The dose was then increased by 0.25 mL every 2 days during a 4‐week titration period until reaching individual maximum tolerated dose based on side effects (notably morning drowsiness), up to a maximum of 3.5 mL per day. This dose was maintained for the subsequent 12 weeks. The titration schedule is detailed in Supporting Information SI2.

Compliance was monitored by weighing returned bottles at each visit and comparing the remaining volume against participant dosing diaries.

### 2.7. Study Duration and Procedures

Each participant was enrolled in the study for a total duration of 20 weeks, consisting of a 4‐week titration phase (to determine the individually tolerated dose), followed by 12 weeks of stable dosing, and concluding with a 4‐week posttreatment follow‐up.

Following the eligibility screening visit, study visits were conducted at enrolment (Week 4), baseline (Week 0, immediately after 4‐week titration) and at Weeks 4, 8 and 12. At each visit, blood samples were collected, questionnaires completed and AEs assessed. Participants also recorded medication use, pain scores and AEs daily in diaries. A final follow‐up phone call was conducted 4 weeks after treatment completion (Week 16).

The detailed trial layout and assessment schedule are provided in Supporting Information SI3.

### 2.8. Primary Outcomes

The primary outcomes of the clinical trial were the feasibility of both the trial procedures and the intervention. The trial’s procedural feasibility was evaluated by examining recruitment and retention metrics, including the ratio of screened to enrolled participants, the difference between projected and actual recruitment rates (accrual rate), retention rates at various stages, completion rates and adherence to planned visits. The intervention’s feasibility was assessed through compliance with the treatment schedule, including missed doses and the number of participants taking the investigational medicinal product (IMP) at least 90% of the time. Participant satisfaction was assessed using selected 5‐point Likert scale questions specifically measuring satisfaction with the product, procedures and overall trial experience from customised participant feedback questionnaire.

Prespecified feasibility benchmarks were retention ≥ 80%, ≥ 80% of participants with IMP adherence ≥ 90% and ≥ 80% completion of planned visits and outcome measures. The planned recruitment target was 36 participants; any shortfall and reasons would be documented.

Blood safety pathology including full blood count (FBC) and electrolytes/liver function tests (E/LFTs) were performed at enrolment and all follow‐up visits to monitor safety. AEs were collected at each visit through participant interviews and diary review. All AEs were categorised by severity and relatedness to the IMP, using Common Terminology Criteria for Adverse Events (CTCAE v5) criteria [20].

### 2.9. Secondary Outcomes

To explore preliminary clinical effects of the intervention on fibromyalgia symptoms, several clinical outcomes were assessed as follows:

1. Fibromyalgia impact was assessed using the Fibromyalgia Impact Questionnaire Revised (FIQR), a 21‐item self‐report measure comprising three domains: function, overall impact and symptoms. Scores range from 0 to 100, with higher scores indicating greater impact [21].
2. Quality of life was measured by the 36‐item Short‐Form Health Survey (SF‐36), which assesses both physical and mental health; scores range from 0 to 100, where higher scores reflect better health [22, 23].
3. Pain was evaluated using the ADPS (7‐day mean) on an 11‐point numeric rating scale (0 representing no pain and 10 the worst pain), based on the worst pain experienced in the past 24 h [18].
4. Depression and anxiety were assessed with the Hospital Anxiety and Depression Scale (HADS), a 14‐item self‐report instrument featuring separate subscales for anxiety and depression (each ranging from 0 to 21); higher scores indicate greater symptom severity [24].
5. Sleep quality was measured using the Pittsburgh Sleep Quality Index (PSQI), a 19‐item questionnaire with seven components; total scores range from 0 to 21, with scores above 5 indicating significant sleep disturbance [25].
6. Fatigue was assessed with the Multidimensional Fatigue Inventory (MFI‐20), a 20‐item scale covering general, physical and mental fatigue as well as motivation and activity levels; items are scored from 0 to 7, where higher scores represent greater fatigue [26].

Timing of outcome assessments is detailed in Supporting Information SI3.

### 2.10. Data Collection

Study data were collected and managed using REDCap electronic data capture tools hosted at Southern Cross University [27, 28].

Screening questionnaires GAD‐7 and PHQ‐9 were completed electronically onsite via REDCap. Additionally, 7‐day ADPSs were collected as part of screening through automated text messages configured through REDCap using Twilio integration [29].

The 4‐week titration phase involved daily electronic self‐assessments delivered via automated text messages, where participants recorded their IMP dose, ADPS and any AEs.

Patient‐Reported Outcome Measures (PROMs)—including the FIQR, MFI, PSQI, HADS and SF‐36—were completed electronically at enrolment (Week 4), baseline (Week 0) and Weeks 4, 8 and 12 visits. Participants also completed an End of Trial Evaluation report at their final visit.

In addition, participants maintained paper diaries to record medication doses, ADPS, AEs and any changes in concomitant medications.

### 2.11. Statistical Analysis

The statistical analysis for the trial was conducted using IBM SPSS Version 29.0 [30].

For the feasibility analyses, all randomised participants were included in the analysis. Basic sociodemographic and clinical data, including comorbidities and medications, were collected at enrolment. These data were analysed to describe the sample characteristics, identify any potential confounding variables that might influence the trial outcomes and ensure comparability between the treatment and placebo groups at enrolment.

Descriptive statistics was used to summarise the primary outcome data as either means (absolute, relative or percentage change) with standard deviations or medians with interquartile range as appropriate for continuous data. Absolute and relative frequencies were used for categorical variables.

Compliance was assessed by calculating the reconciliation difference between the participant‐recorded consumption and the actual consumption based on bottle weights, as well as the percentage of participants taking the IMP at least 90% of the time. Participants exceeding the 10% threshold were to be considered noncompliant and excluded from the analysis. Median IMP doses were reported for each group. AEs were summarised by severity and relatedness to the IMP.

The statistical analyses for secondary outcomes were conducted on a subset of participants, excluding those who withdrew or had invalid data. A complete case approach was used with no imputation of missing data, appropriate for this feasibility trial design. Between‐group and within‐group comparisons were conducted across multiple measures: ADPS, PSQI, FIQR, SF‐36, MFI‐20 and HADS.

For weekly ADPS, within‐group changes from enrolment to Week 1 (post‐titration) and to Week 12 (end of treatment) were assessed using paired samples *t*‐tests, with results presented as two‐tailed *p* values with means (SD). Between‐group differences at Week 1 and Week 12 were analysed using univariate analysis of covariance (ANCOVA), adjusting for values at enrolment.

For FIQR, PSQI, SF‐36, MFI‐20 and HADS (collected every four weeks), within‐group changes were assessed using paired samples *t*‐tests from enrolment to Week 12. Between‐group differences for these measures were reported at Week 12, using ANCOVA adjusted for values at enrolment.

Between‐group results were reported as mean differences with 95% confidence intervals (CIs). To evaluate the magnitude of treatment effects, partial eta squared (*η* ^2^ *p*) was reported and interpreted as small (≈ 0.01), medium (≈ 0.06) and large (≈ 0.14), where a small effect indicates a minor impact, a medium effect suggests a moderate and potentially clinically relevant impact and a large effect reflects a strong and substantial treatment effect [31–33]. Given the small sample size, emphasis was placed on effect sizes rather than *p* values when interpreting between‐group differences [34–36].

Additionally, the study reported the proportion of patients achieving clinically meaningful improvements, including the minimum clinically important difference (MCID) for FIQR at Week 12, where a 45.5% improvement on the total FIQR score represents the MCID [37], and clinically meaningful pain reductions of ≥ 30% and substantial reductions of ≥ 50% for ADPS scores assessed at both Week 1 and Week 12 [38].

## 3. Results

### 3.1. Participant Flow

**FIGURE 1:**
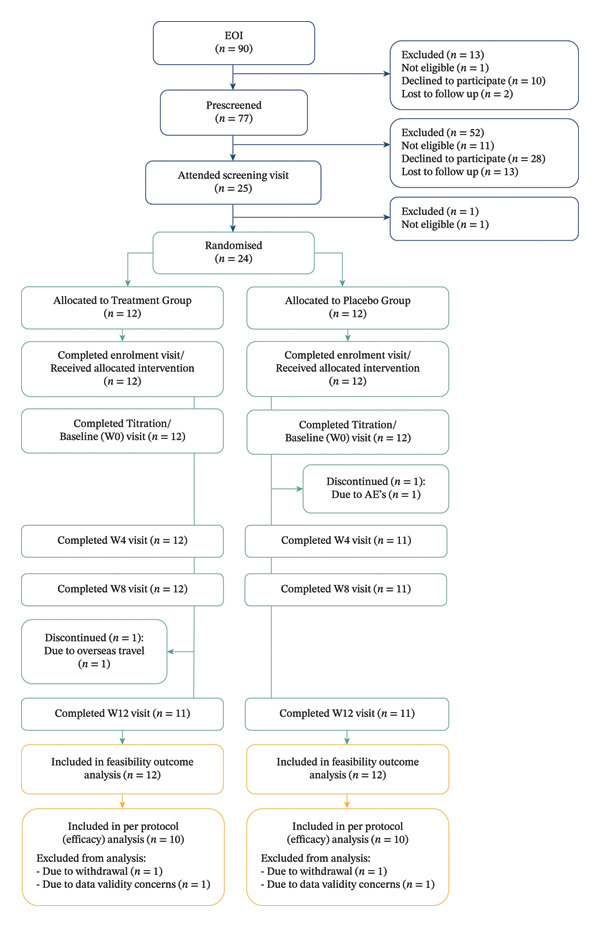
CONSORT flow diagram.

A total of 90 people expressed interest in participating in the study, of whom 77 completed prescreening (see Figure 1 for CONSORT diagram). Twenty‐four participants were randomised (12 per group), of whom 22 completed the trial (11 per group). Primary feasibility outcomes were analysed using all randomised participants (*n* = 24). Secondary clinical outcomes were analysed using participants who completed the trial with valid data (*n* = 20; 10 per group), as detailed in Figure 1. Recruitment took place over approximately six months, from August 2023 to January 2024, followed by a treatment and follow‐up period concluding in June 2024.

### 3.2. Primary Outcomes

#### 3.2.1. Recruitment and Retention

Twelve people were found ineligible during expression of interest and prescreening stages: nine were excluded due to comorbidities, including rheumatoid arthritis (3), psoriatic arthritis (1), ankylosing spondylitis (1), recent cancer (1), neurologic (1) and mental health conditions (2). One individual was deemed ineligible by the principal investigator due to multiple concomitant medications, one was excluded due to a recent history of drug or alcohol abuse and one did not have a confirmed diagnosis of fibromyalgia.

Approximately every third prescreened individual progressed to the screening visit (*n* = 25). One person was excluded at this stage due to a history of drug‐induced psychosis. All participants underwent drugs of abuse testing at screening. No participants required a cannabis washout period, as all tested negative for THC at both baseline screening and prior to randomisation. Twenty‐four people were found to be eligible for participation and randomised into either the treatment or placebo group. Upon unblinding, it was found that *n* = 12 were assigned to the treatment group and *n* = 12 to the placebo group. Of 77 participants who completed prescreening, 24 were randomised, yielding a screening‐to‐enrolment ratio of approximately 3:1 (31.2%).

The trial recruited 12 fewer participants than projected which resulted in an accrual rate of 66.67%. The main recruitment barriers were legal concerns about cannabis use and driving and geographic limitations, with 6 of 38 people who declined participation citing legal issues and 14 citing travel distance. The recruitment period was not extended due to budget exhaustion and the constraints of the first author’s PhD candidature timeline.

The completion rate was 91.67%, with 22 out of 24 randomised participants completing the trial. Two participants discontinued the trial early: one treatment group participant due to travelling to a country where medicinal cannabis was not permitted and one placebo participant due to adverse reactions.

Against prespecified feasibility benchmarks, retention (91.67%) and adherence (100% of participants with ≥ 90% IMP compliance; see Section 3.2.2) met targets. All planned visits were completed (with only a few requiring rescheduling), and outcome measure completion exceeded the ≥ 80% benchmark. Recruitment did not meet the planned target of 36 participants within the defined recruitment window.

Both groups were well matched in terms of demographics and baseline characteristics such as age, sex, duration of fibromyalgia symptoms, time since diagnosis and various health metrics like weight, height, BMI, blood pressure, pain levels, depression and anxiety scores (Table 1). Participants were predominantly Caucasian females in their early to mid‐50 s, married or divorced and had been experiencing fibromyalgia symptoms for about 13–14 years but were diagnosed approximately 6 years ago. The average participant was overweight or obese (BMI around 30 kg/m^2^) with mild depression and anxiety symptoms; their blood pressure and heart rate were within normal ranges.

**TABLE 1:** Participants baseline characteristics.

Participants baseline characteristics	Treatment group (*n* = 12)	Placebo group (*n* = 12)
Age, in years	55.25 (13.7)	52.25 (13.16)
Sex (female), *N* (%)	12 (100%)	12 (100%)
Fibromyalgia status		
Duration of FMS symptoms, in years	13.63 (17.2)	13.79 (12.2)
Duration of FMS since diagnosis, in years	5.68 (5.7)	6.46 (7.8)
Ethnicity, *N* (%)		
Caucasian	11 (91.7%)	12 (100%)
Other	1 (8.3%)	0 (0%)
Marital status, *N* (%)		
Married	6 (50.0%)	5 (41.7%)
De facto	2 (16.7%)	1 (8.3%)
Divorced	3 (25.0%)	4 (33.3%)
Never married	1 (8.3%)	2 (16.7%)
Blood pressure		
Systolic, in mmHg	127.33 (15.1)	127.75 (17.7)
Diastolic, in mmHg	78.67 (6.9)	80.58 (7.0)
Heart rate, in beats per min	74.42 (12.4)	81.17 (11.6)
Height, in m	1.63 (0.06)	1.64 (0.06)
Weight, in kg	79.07 (16.2)	80.87 (16.0)
BMI, in kg/m^2^	30.04 (6.8)	30.14 (5.6)
7‐Day ADPS, 0–10	6.02 (1.2)	6.32 (0.9)
Total PHQ‐9 (Depression) score, 0–27	7.42 (3.3)	8.42 (2.4)
Total GAD‐7 (Anxiety) score, 0–21	4.00 (3.4)	4.50 (2.2)

*Note:* Data are presented as means (SD) unless otherwise specified.

#### 3.2.2. Compliance

All participants took the IMP (either cannabis or placebo) at least 90% of the time, with an overall missed dose rate of 1.2%. Of the 24 participants, 11 (45.8%) did not miss any doses. The participant with the highest missed dose rate of 5.4% remained included in the analysis, as this did not exceed the 10% noncompliance threshold.

Overall, the reconciliation rate was 68.8%, with 85.1% in the treatment group and 52.2% in the placebo group. Discrepancies were primarily due to spillage and administration errors in the treatment group and missed doses or broken syringe stoppers in the placebo group. Participants on doses ≤ 1.5 mL had a higher reconciliation rate (90.6%) compared with those on > 1.5 mL (40.0%).

#### 3.2.3. Titration, Drug Dose and Tolerability

The 4‐week titration phase was generally well tolerated and effective in achieving individual IMP doses, except for two participants. For one participant, dose reduction post‐titration was required due to persistent morning drowsiness, while for another participant, it was required due to daytime sleepiness, increased fatigue, lethargy and memory issues.

In the treatment group, final doses ranged from 0.5 to 3.5 mL (median of 1 mL), while in the placebo group, final doses ranged from 0.25 to 3.5 mL (median of 2.25 mL). Average weekly doses for both groups during titration are presented in Supporting Information SI4.

The study drug was well tolerated by participants, with most AEs being mild, and no severe or life‐threatening events were observed in either group. Notably, one placebo participant withdrew from the trial due to AEs, including somnolence, cognitive disturbances, urticaria and pruritus.

#### 3.2.4. Safety

**FIGURE 2:**
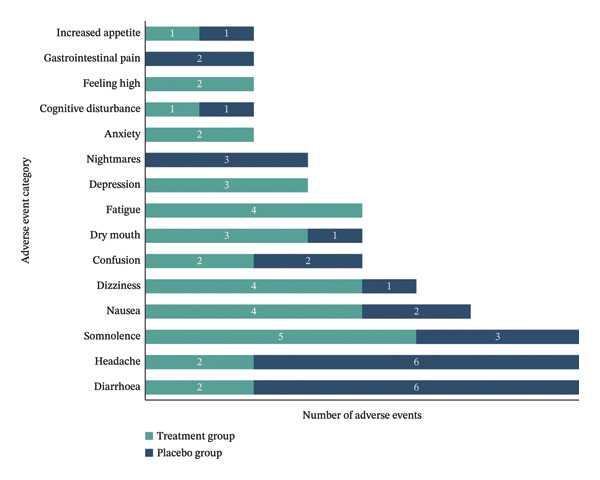
Adverse events count by category, possibly/probably related to IMP, by group. Total count of adverse events during the trial in each AE category that were judged possibly or probably related to the IMP, reported separately for the treatment and placebo groups. Counts reflect events, not unique participants; individuals may experience multiple events.

A total of 121 AEs were reported by all participants, with the majority being mild and most considered possibly or probably related to the IMP. Further details on AE frequency, relatedness and severity by treatment group are presented in Table 2. Figure 2 shows the frequency of the most common AEs possibly or probably related to IMP for both treatment and placebo groups.

**TABLE 2:** Adverse event summary by group.

Characteristic	Treatment group (*n* = 12)	Placebo group (*n* = 12)
Total number of AEs	62	59
Number of AEs per participant:		
Median (range)	4.5 (2–11)	3.0 (1–20)
Most common AEs (*n*)	Somnolence (5)	Headache (9)
	Dizziness (5)	Diarrhoea (6)
	Fatigue (5)	Nausea (5)
	Nausea (4)	Flu‐like symptoms (4)
	Dry mouth (4)	Somnolence (3)
Relatedness to IMP:		
Not related + Unlikely related, *n* (%)	16 (25.8%)	19 (32.2%)
Possibly + Probably related, *n* (%)	46 (74.2%)	40 (67.8%)
Severity:		
Mild, *n* (%)	58 (93.5%)	47 (79.7%)
Moderate, *n* (%)	4 (6.5%)	12 (20.3%)
Severe/life threatening, *n* (%)	0 (0%)	0 (0%)

No significant changes were observed in BMI, blood pressure, FBC or E/LFTs in either group throughout the trial.

#### 3.2.5. Satisfaction

Participant satisfaction was high across both groups, as assessed at the end of the trial. Product satisfaction was greater in the treatment group (75%) compared with the placebo group (41.7%), and a majority in the treatment group (83.3%) indicated they would recommend the investigational product, compared with 41.7% in the placebo group. Additionally, 75% of the participants in each group correctly guessed their allocation.

### 3.3. Secondary Outcomes

Detailed summaries of secondary outcomes including group means, between‐group differences with 95% CIs and effect sizes are presented in Tables 3 and 4. One placebo participant had incomplete SF‐36 Role‐Physical and Role‐Emotional data at one or more time points, resulting in *n* = 9 for these specific domains and component summary scores.

**TABLE 3:** Pain reduction outcomes (ADPS) comparing treatment and placebo groups.

Timepoint	Within‐group changes		Between group difference		MCID responders	
	Treatment (*n* = 10)	Placebo (*n* = 10)	Mean difference, (95% CI)	Effect size (*η* ^2^ *p*)	Clinically meaningful ≥ 30% pain reduction	Substantial improvement ≥ 50% pain reduction
Enrolment	6.00 (1.33)	6.34 (0.96)				
Week 1	3.50 (1.72), *p* < 0.001	5.16 (1.84), *p* = 0.073	−1.41 (−2.95–0.13)	0.180 (large)	Treatment 70% vs. Placebo 20%.	Treatment 40% vs. Placebo 10%
Week 12	3.83 (1.60), *p* = 0.001	5.05 (2.11), *p* = 0.076	−1.02 (−2.73–0.69)	0.085 (medium)	Treatment 70% vs. Placebo 40%.	Treatment 20% vs. Placebo 20%

*Note:* ADPS*:* Average Daily Pain Score; higher scores indicate greater pain severity. Within‐group changes represent mean (SD) pain scores at enrolment, and Week 1 and Week 12 timepoints, with paired *t*‐test *p* values for change from enrolment; *p* values correspond to statistical significance of within‐group changes over time. Between‐Group Differences at Week 1 or Week 12 (95% CI) show the difference in mean difference between treatment and placebo groups with 95% confidence intervals. Effect Size *(* *η* ^2^ *p*) indicates the magnitude of between‐group differences, with small (< 0.06), medium (0.06–0.14), and large (> 0.14) effect sizes. MCID Responders denote the proportion of participants achieving clinically meaningful pain reductions of ≥ 30% and ≥ 50%, respectively.

**TABLE 4:** Summary of secondary outcome measures including sleep quality, function, fatigue, mental health and quality of life.

Outcome measure	Within‐group changes						Between‐group differences at Week 12		MCID responders
	Treatment (*n* = 10)^∗^			Placebo (*n* = 10)^∗^					
	Enrolment	Week 12	Mean difference (95% CI), *p* value	Enrolment	Week 12	Mean difference (95% CI), *p* value	Mean difference, (95% CI)	Effect size (*η* ^2^ *p*)	
*Sleep Quality (PSQI)*									
Total score (0–21)	13.38 (2.53)	8.50 (3.27)	−4.88 (−6.78–−2.98), *p* < 0.001	13.54 (4.69)	11.65 (4.12)	−1.89 (−5.63–1.85), *p* = 0.283	−3.09 (−6.42–0.25)	0.184 (large)	N/A

*Fibromyalgia Impact (FIQR)*									
Function domain (0–90)	43.30 (19.35)	24.90 (18.14)	−18.40 (−31.51–−5.29), *p* = 0.011	54.10 (20.63)	43.00 (21.69)	−11.10 (−26.43–4.23), *p* = 0.136	−12.67 (−30.11–4.78)	0.121 (medium)	N/A
Impact domain (0–20)	11.40 (3.37)	5.60 (4.58)	−5.80 (−10.01–−1.59), *p* = 0.012	12.90 (3.87)	10.10 (4.86)	−2.80 (−6.70–1.10), *p* = 0.139	−4.31 (−8.98–0.36)	0.183 (large)	N/A
Symptoms domain (0–100)	51.70 (6.96)	36.80 (19.65)	−14.90 (−25.88–−3.92), *p* = 0.013	60.70 (12.27)	52.80 (18.21)	−7.90 (−20.19–4.39), *p* = 0.180	−7.26 (−24.77–10.24)	0.043 (small)	N/A
Total score (0–100)	51.68 (8.08)	32.30 (16.98)	−19.38 (−31.06–−7.71), *p* = 0.005	61.28 (15.35)	50.83 (19.02)	−10.45 (−24.17–3.27), *p* = 0.119	−13.48 (−31.16–4.19)	0.132 (large)	≥ 45.5% reduction: Treatment 40% vs. Placebo 10%

*Fatigue (MFI-20)*									
General fatigue (4–20)	11.20 (1.40)	11.00 (0.67)	−0.20 (−1.26–0.86), *p* = 0.678	11.80 (0.92)	11.60 (1.43)	−0.20 (−1.41–1.01), *p* = 0.716	−0.57 (−1.69–0.55)	0.064 (medium)	N/A
Physical fatigue (4–20)	13.30 (1.42)	13.50 (1.35)	0.20 (−1.31–1.70), *p* = 0.770	12.70 (1.25)	12.90 (1.45)	0.20 (−0.86–1.26), *p* = 0.678	0.52 (−0.87–1.91)	0.036 (small)	N/A
Activity (4–20)	12.70 (1.77)	12.00 (1.05)	−0.70 (−1.82–0.42), *p* = 0.191	12.90 (1.10)	12.20 (1.48)	−0.70 (−1.66–0.26), *p* = 0.132	−0.12 (−1.24–0.99)	0.003 (small)	N/A
Motivation (4–20)	11.00 (2.91)	10.90 (2.47)	−0.10 (−2.30–2.10), *p* = 0.920	11.00 (3.62)	11.20 (2.94)	0.20 (−2.34–2.74), *p* = 0.863	−0.30 (−2.72–2.12)	0.004 (small)	N/A
Mental fatigue (4–20)	10.80 (1.55)	11.20 (1.03)	0.40 (−0.87–1.67), *p* = 0.494	11.30 (0.82)	11.30 (1.06)	0.00 (−0.89–0.89), *p* = 1.000	−0.05 (−1.09–0.98)	0.001 (small)	N/A

*Mental Health (HADS)*									
Anxiety (0–21)	7.00 (4.64)	6.20 (4.47)	−0.80 (−2.18–0.58), *p* = 0.223	9.40 (2.91)	9.60 (3.69)	0.20 (−2.45–2.85), *p* = 0.868	−1.56 (−4.43–1.32)	0.071 (medium)	N/A
Depression (0–21)	7.12 (3.08)	6.00 (4.06)	−1.12 (−3.15 to 0.91), *p* = 0.245	8.40 (2.01)	8.90 (3.25)	0.50 (−1.89–2.89), *p* = 0.647	−1.89 (−4.95 to 1.17)	0.091 (medium)	N/A

*Quality of life (SF-36)*									
Physical functioning (0–100)	37.22 (24.13)	49.50 (27.13)	12.28 (2.43–22.13), *p* = 0.020	25.50 (22.17)	30.50 (20.34)	5.00 (−8.90–18.90), *p* = 0.437	9.96 (−6.10–26.02)	0.091 (medium)	N/A
Role‐Physical (0–100)^∗^	22.50 (41.58)	35.00 (33.75)	12.50 (−26.36–51.36), *p* = 0.485	0.00 (0.00)	12.50 (27.00)	12.50 (−6.82–31.82), *p* = 0.177	23.04 (−8.94–55.03)	0.120 (medium)	N/A
Bodily pain (0–100)	29.50 (9.90)	49.60 (14.80)	20.10 (6.01–34.19), *p* = 0.010	26.30 (15.00)	33.70 (18.07)	7.40 (−11.14–25.94), *p* = 0.390	16.84 (1.09–32.60)	0.230 (large)	N/A
General health perceptions (0–100)	49.80 (17.11)	44.30 (19.65)	−5.50 (−14.77–3.77), *p* = 0.212	33.95 (24.71)	32.40 (22.51)	−1.55 (−6.89–3.79), *p* = 0.528	−1.90 (−12.55–8.75)	0.008 (small)	N/A
Vitality (0–100)	26.50 (11.80)	42.00 (18.74)	15.50 (4.78–26.22), *p* = 0.010	17.00 (16.36)	28.33 (28.51)	11.33 (−12.46–35.13), *p* = 0.309	10.87 (−13.58–35.31)	0.049 (small)	N/A
Social functioning (0–100)	47.50 (26.22)	62.50 (22.82)	15.00 (1.15–28.85), *p* = 0.037	33.75 (21.29)	37.50 (22.05)	3.75 (−14.16–21.66), *p* = 0.647	18.09 (−1.15–37.33)	0.188 (large)	N/A
Role‐Emotional (0–100)^∗^	36.67 (45.68)	53.33 (45.00)	16.67 (−24.25–57.58), *p* = 0.381	46.67 (39.13)	43.33 (47.27)	−3.33 (−20.93–14.26), *p* = 0.678	15.53 (−23.32–54.38)	0.040 (small)	N/A
Mental health (0–100)	66.40 (17.81)	72.40 (20.26)	6.00 (−3.27–15.27), *p* = 0.177	67.20 (9.76)	61.20 (18.29)	−6.00 (−16.47–4.47), *p* = 0.227	11.95 (−1.45–25.35)	0.172 (large)	N/A
Physical component summary^∗^	29.44 (8.37)	33.50 (7.51)	4.06 (−1.70–9.82), *p* = 0.146	21.78 (8.48)	25.88 (5.65)	4.10 (−1.47–9.68), *p* = 0.130	4.80 (−1.53–11.12)	0.131 (medium)	N/A
Mental component summary^∗^	42.59 (11.68)	47.47 (12.22)	4.88 (−1.02–10.78), *p* = 0.094	43.16 (7.02)	42.07 (14.67)	−1.58 (−8.11–4.95), *p* = 0.598	6.54 (−1.89–14.97)	0.136 (medium)	N/A

*Note:* Within‐group values are reported as mean (standard deviation) with within‐group difference (95% confidence interval) and *p* value from enrolment to Week 12; *p* values correspond to statistical significance of within‐group changes over time. *Between-group differences at Week 12 (95% CI)* represent the difference in mean change between treatment and placebo groups with corresponding confidence intervals. *Effect size (* *η* ^2^ *p*) indicates the magnitude of group differences; interpreted as small (< 0.06), medium (0.06–0.14), or large (> 0.14). *MCID Responders* show the proportion of participants achieving minimal clinically important differences where reported. PSQI = Pittsburgh Sleep Quality Index (lower scores indicate better sleep quality); FIQR = Fibromyalgia Impact Questionnaire Revised (lower scores indicate better function/less impact); MFI‐20 = Multidimensional Fatigue Inventory (lower scores indicate less fatigue); HADS = Hospital Anxiety and Depression Scale (lower scores indicate fewer symptoms); SF‐36 = Short Form Health Survey (higher scores indicate better health‐related quality of life).

^∗^Placebo group *n* = 9 for SF‐36 Role‐Physical, Role‐Emotional and component summary scores due to missing data at one or more timepoints for one participant. All other measures: Treatment *n* = 10 and Placebo *n* = 10.

#### 3.3.1. Pain (ADPS)

**FIGURE 3:**
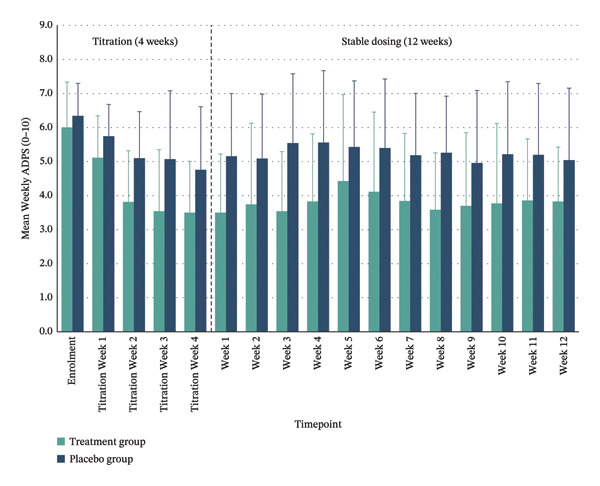
Weekly Average Daily Pain Scores (ADPSs) by group. Mean weekly ADPS represents the average of daily pain scores collected over each 7‐day period. The plot shows group means for the treatment and placebo groups across the 4‐week titration phase followed by the 12‐week maintenance phase. Error bars represent ± standard error of the mean (SE).

Between‐group comparisons adjusted for values at enrolment showed medium to large effect sizes favouring the treatment group for pain reduction at post‐titration (Week 1) (*η* ^2^ *p* = 0.180; 95% CI: −2.95–0.13) and Week 12 (*η* ^2^ *p* = 0.085; 95% CI: −2.73–0.69). Clinically meaningful pain relief—defined as a ≥ 30% reduction in ADPS—was achieved by 70% of the treatment group at both time points, compared with 20% and 40% in the placebo group, respectively. Substantial pain relief (≥ 50% reduction) was observed in 40% of the treated participants post‐titration and 20% at Week 12 versus 10% and 20% in placebo. Within the treatment group, significant reductions in mean pain scores were maintained throughout the trial period, whereas the placebo group showed smaller, nonsignificant changes (Figure 3 and Table 3).

#### 3.3.2. Sleep Quality (PSQI)

At Week 12, a large between‐group effect size favoured treatment for sleep quality improvement (*η* ^2^ *p* = 0.184; 95% CI: −6.42–0.25), with mean PSQI scores decreasing from 13.38 ± 2.53 at enrolment to 8.50 ± 3.27 at Week 12 (*p* < 0.001). In contrast, the placebo group showed minimal changes (Table 3).

#### 3.3.3. Fibromyalgia Impact (FIQR)

Between‐group analyses revealed medium to large effect sizes favouring treatment for the FIQR Function domain (*η* ^2^ *p* = 0.121; 95% CI: −30.11–4.78), Impact domain (*η* ^2^ *p* = 0.183; 95% CI: −8.98–0.36) and total score (*η* ^2^ *p* = 0.132; 95% CI: −31.16–4.19). Clinically meaningful improvement—defined as achieving the MCID of ≥ 45.5% reduction in FIQR total score from enrolment to Week 12—was seen in 40% of the treatment group compared with 10% in placebo. Within‐group improvements across all FIQR domains and total score were significant only in the treatment group (Figure 4 and Table 4).

FIGURE 4Mean FIQR scores at enrolment, baseline (Week 0) and Week 12 by group. Bar graphs showing mean scores for each FIQR domain and total score at enrolment, baseline (Week 0) and Week 12 for treatment and placebo groups: (a) Function domain (0–90); (b) Impact domain (0–20); (c) Symptoms domain (0–100) and (d) Total score (0–100). Data are presented across the 4‐week titration and 12‐week maintenance phases, illustrating changes in fibromyalgia impact over time within each group. Error bars represent ± standard error of the mean (SE).

**Figure:**
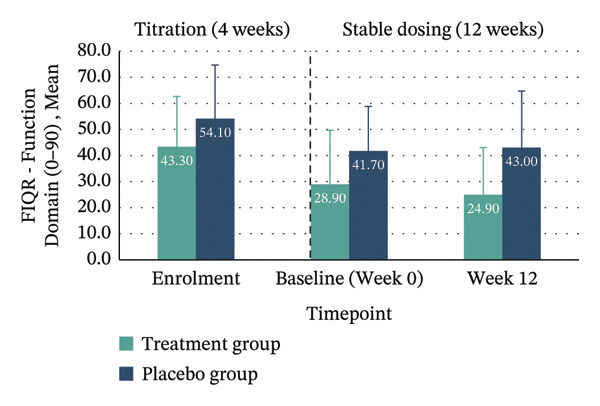
(a)

**Figure:**
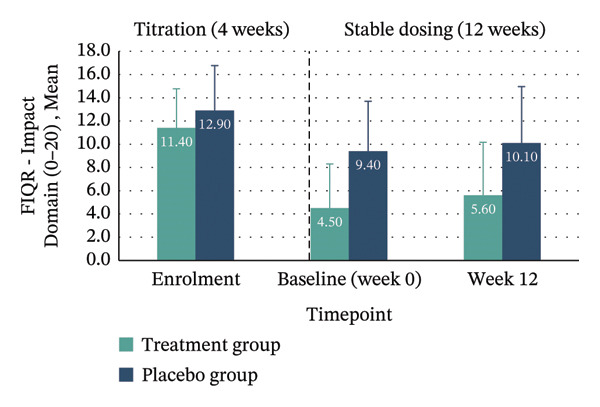
(b)

**Figure:**
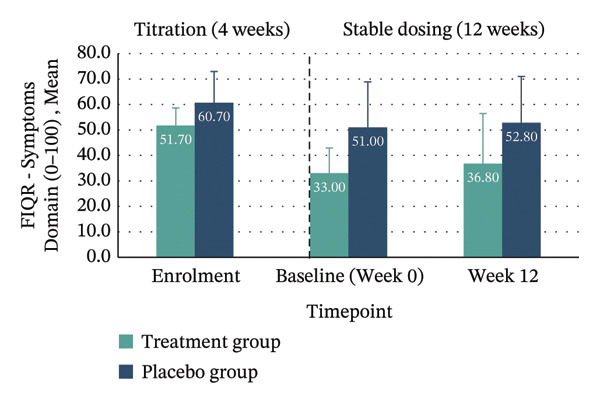
(c)

**Figure:**
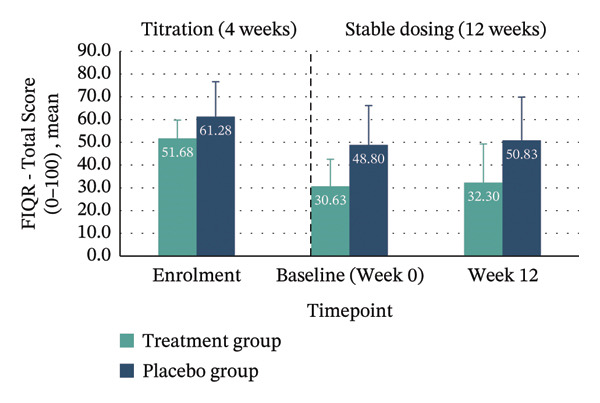
(d)

#### 3.3.4. Quality of Life (SF‐36)

Between‐group effect sizes at Week 12 for SF‐36 domains were large for bodily pain (*η* ^2^ *p* = 0.230; 95% CI: 1.09–32.60), social functioning (*η* ^2^ *p* = 0.188; 95% CI: −1.15–37.33) and mental health (*η* ^2^ *p* = 0.172; 95% CI: −1.45–25.35), favouring the treatment group. Significant within‐group improvements in physical functioning, bodily pain, vitality and social functioning were observed only in the treatment group (Table 4).

#### 3.3.5. Fatigue (MFI‐20) and Anxiety/Depression (HADS)

Medium between‐group effect sizes favoured the treatment group for anxiety and depression scores (HADS), as well as for general fatigue measured by the MFI‐20, while no notable effects were observed in the other MFI‐20 fatigue domains. No statistically significant within‐group changes were observed for these measures (Table 4).

## 4. Discussion

This feasibility trial evaluated an oral medicinal cannabis formulation containing a 1:1 ratio of THC and CBD (10 mg/mL each) for fibromyalgia, providing insights into the feasibility of cannabis‐based clinical research in this population, as well as preliminary evidence of therapeutic benefits.

### 4.1. Recruitment

Recruitment was facilitated by strong interest from potential participants eager to access medicinal cannabis as a treatment option. However, the study fell short of the planned 36 participants, mainly due to geographic barriers and legal concerns. Many candidates declined because of the need to travel to the single study site, reflecting common challenges in single‐location trials requiring physical visits that limit rural and remote participation [39]. Additionally, legal concerns significantly impacted willingness to participate due to Australia’s, and specifically Queensland’s, strict ‘zero tolerance’ policy for THC detection in drivers, which criminalises any detectable THC regardless of impairment or medicinal use [40, 41]. As a result, many potential candidates declined participation out of concern for legal consequences from roadside drug testing. This contrasts with other Australian jurisdictions such as Victoria and Tasmania, which have lifted driving restrictions for prescribed medicinal cannabis users provided they are unimpaired [42, 43]. Similar regulatory approaches exist in several European countries (Germany, United Kingdom, Ireland and Norway), where prescribed users may drive if unimpaired, with emphasis placed on functional impairment rather than THC presence [40]. Canada applies per se blood THC limits but prioritises impairment for prosecution; some provinces add restrictions for specific driver groups [44–46]. In the United States, state‐level variation includes per se limits, impairment requirements or exemptions for medicinal users [47, 48].

To improve recruitment in cannabis trials in Australia and other countries with similarly restrictive THC driving laws, policy reform is needed to adopt impairment‐based limits, legally protect prescribed trial participants and provide clear clinical guidance [40, 41]. Future studies should also consider decentralised or hybrid designs using telemedicine, mobile clinics and local healthcare collaborations to improve accessibility and diversity [39].

### 4.2. Retention and Compliance

Retention was high, with 22 of the 24 participants completing the trial (92%), suggesting strong participant acceptance of both the intervention and study procedures. Medication compliance was similarly high; all participants took at least 90% of their doses with minimal missed doses, reflecting positive patient expectations around cannabis as a natural and effective treatment for chronic pain [49, 50]. Structured clinical guidance during the 4‐week titration likely supported adherence by allowing personalised dose optimisation and side‐effect management [50].

The 4‐week titration phase enabled participants to find an optimal dose gradually, reducing the risk of AEs commonly seen with fixed‐dose protocols [19, 51–54]. However, dosing challenges emerged at higher volumes (> 1.5 mL), including dosing errors such as spillages and difficulties using the 1‐mL syringe—requiring multiple draws—increasing discrepancies. These findings suggest future studies should optimise administration methods and explore alternative delivery options to improve ease of use, tolerability and data accuracy.

### 4.3. Preliminary Efficacy

Although exploratory efficacy assessment was a secondary focus, the treatment group showed promising improvements across fibromyalgia symptoms, including pain, sleep quality, quality of life and fibromyalgia impact parameters with medium to large effect sizes. These preliminary findings should be interpreted cautiously given the small sample and wide CIs. Nonetheless, the observed improvements align with the endocannabinoid system’s role in modulating pain, mood and sleep—key dysregulated domains in fibromyalgia [7–9]—and support prior evidence for cannabinoids’ therapeutic potential in this condition [10].

The observed symptom improvements were consistent with previous research in fibromyalgia cohorts employing various THC:CBD ratios and delivery methods [10, 54–56]. In particular, reductions in severity of pain, the hallmark symptom of fibromyalgia [17, 57], were clinically meaningful, reinforcing the analgesic effects of cannabis demonstrated in both randomised controlled trials and observational studies [10, 54–56]. Similarly, notable improvements in sleep quality further support cannabis’s potential to address common fibromyalgia‐related sleep disturbances, complementing findings from other clinical trials [51, 54, 58].

Beyond symptom relief, functional capacity and overall quality of life also improved with treatment, reflecting the broader impact of medicinal cannabis on daily living and wellbeing. These multidimensional benefits resonate with the systematic review and prior observational data indicating positive effects on physical functioning, vitality and social engagement among fibromyalgia patients treated with medicinal cannabis [10, 55, 58].

For fatigue and mood symptoms, medium effect sizes were observed for anxiety, depression and general fatigue, though results were mixed; fatigue measures showed variable directionality across domains, and some treatment group participants reported fatigue, anxiety or depression as AEs (Figure 2) while others experienced improvements. This variability highlights substantial individual differences in response to cannabinoids for psychological symptoms. The small sample size limits conclusions, but these findings underscore the importance of identifying both positive and negative responders in future trials. Further research with larger samples and detailed phenotyping is needed to clarify how different cannabinoid profiles affect psychological symptoms in fibromyalgia, considering CBD’s anxiolytic and THC’s psychoactive properties [59].

### 4.4. Safety

The safety profile was encouraging; most AEs were mild and consistent with known effects of cannabis‐based treatments (somnolence, nausea, dizziness, fatigue and dry mouth) [10]. No serious AEs occurred, and laboratory tests remained stable. Compared with many pharmacological treatments for fibromyalgia with higher toxicity and dropout rates [60], medicinal cannabis appears comparatively well tolerated.

The 4‐week titration phase effectively helped most participants with establishing optimal doses while managing side effects, thus supporting personalised gradual titration as best practice for cannabinoids [19]. Two participants required dose reductions post‐titration due to persistent central nervous system effects like drowsiness and cognitive disturbances, highlighting significant individual variability and the need for careful monitoring throughout dosing to optimise safety and tolerability [19].

### 4.5. Strengths and Limitations

The study employed a rigorous randomised, double‐blind, placebo‐controlled design with comprehensive safety monitoring and multiple validated PROMs. Unlike many earlier observational or open‐label studies [52, 54, 55, 58] and a randomised trial using inhaled cannabis [61] conducted in fibromyalgia cohorts, this trial used oral administration of 1:1 THC:CBD cannabis oil, allowing precise dosing and gradual 4‐week individualised titration. This administration method potentially improves safety by reducing rapid psychoactive peaks typical of inhaled cannabis [19, 62] and aligns with pharmaceutical standards addressing variability in inhaled delivery [51, 63]. The structured titration phase enabled optimal dosing and side‐effect management, refining prior fixed‐dose protocols [51–54]. Feasibility metrics including recruitment, retention, compliance and dosing accuracy provided essential groundwork for larger efficacy trials [64]. Additionally, the use of digital platforms for daily symptom and AE reporting enhanced data completeness and engagement [28].

Certain limitations are inherent to feasibility trials, such as the small sample size. This study enrolled 24 participants, which aligns with commonly recommended sample sizes for feasibility trials but still limits statistical power and generalisability; therefore, the efficacy results should be interpreted in this context [65, 66]. However, some limitations are specific to this study’s design and context. Recruitment was constrained by geographic barriers and Queensland’s strict THC driving laws, which likely reduced willingness to participate. The single‐site design may limit applicability to more diverse populations. The 4‐week titration and 12‐week treatment duration may be insufficient to assess long‐term safety and efficacy. Importantly, 75% of the participants in both groups correctly guessed their treatment allocation, likely due to the distinctive psychoactive effects of THC [67]. Despite implemented blinding procedures, this high rate of correct allocation guessing may have introduced expectation bias on subjective patient‐reported outcomes [68]. This challenge is not unique to the present trial—a recent Australian randomised controlled trial of oral THC/CBD for insomnia reported a similarly high correct allocation guessing rate (70%) following a single dose in a controlled laboratory setting [69]—and reflects a well‐documented inherent limitation of blinding in THC‐containing cannabis trials [67, 70]. All outcomes except blood markers were self‐reported and therefore particularly sensitive to such expectation effects. The substantial placebo response observed in this study (40% achieving ≥ 30% pain reduction at Week 12) is consistent with high placebo effects typical of fibromyalgia trials, where placebo response rates can exceed 30%–40% and occasionally rival active treatment responses [71].

Additionally, the enrolled sample was 100% female, which occurred incidentally rather than by design. This restricts generalisability given documented sex differences in endocannabinoid system biology, pain processing and cannabinoid pharmacokinetics [72, 73].

### 4.6. Future Directions

Future research should prioritise overcoming recruitment barriers by adopting decentralised or hybrid trial designs leveraging telemedicine consultations, mobile clinic visits and partnerships with local healthcare providers to increase accessibility for rural or mobility‐limited populations [39]. Concurrently, advocacy for regulatory reform is important to address legal obstacles such as restrictive THC driving laws that currently limit patient participation [40, 41]. To better distinguish true pharmacological effects from placebo responses in the presence of high placebo effects typical of chronic pain trials [74], future studies could employ crossover designs (which control for individual placebo variability) or enrichment strategies such as open‐label run‐ins to identify and exclude high placebo responders prior to randomised, blinded phases [75]. Larger‐scale confirmatory trials with adequate power are needed to robustly assess efficacy across fibromyalgia’s diverse symptom domains [76]. Optimisation of drug delivery methods is warranted to improve dosing accuracy and ease of administration. Additionally, further investigation into the differential effects of various cannabinoid profiles on psychological symptoms is warranted, given mixed evidence on mood outcomes and the distinct pharmacological actions of THC versus CBD [59].

## 5. Conclusion

This randomised, double‐blind, placebo‐controlled feasibility study of 1:1 THC:CBD (10 mg/mL each) cannabis oil in fibromyalgia showed high retention and adherence, while recruitment fell short of targets under the study’s constraints. The intervention appeared well tolerated with no serious AEs in this small sample. Secondary outcomes suggest potential benefits in pain, sleep, function and quality of life, though findings should be interpreted cautiously given the small sample and wide confidence intervals. Larger, adequately powered trials with strategies to address recruitment barriers are needed to confirm efficacy and refine dosing.

## Author Contributions

Jennifer Ng and Gerald Tracey conceived the concept and protocol summary with Janet Schloss and Evelin Tiralongo. Inna Kurlyandchik, Janet Schloss and Evelin Tiralongo designed the study. Jennifer Ng, Gerald Tracey and Romy Lauche contributed to the study design. Gerald Tracey served as principal investigator. Inna Kurlyandchik coordinated the study, conducted study visits and collected data. Janet Schloss assisted with study visits and data collection. Inna Kurlyandchik and Romy Lauche performed statistical analyses. Romy Lauche, Janet Schloss and Evelin Tiralongo verified the data presented in the manuscript. Inna Kurlyandchik drafted the manuscript, with Romy Lauche, Janet Schloss and Evelin Tiralongo contributing to revisions. All authors accessed study data and contributed to manuscript revision.

## Funding

Inna Kurlyandchik’s PhD scholarship was jointly funded by Southern Cross University (SCU) and Little Green Pharma Ltd (LGP), enabling Inna Kurlyandchik to conduct this study. Additional support for trial conduct was provided by Dr Janet Schloss’s personal research funds and PhD allocation funds from the SCU Faculty of Health. LGP supplied the investigational medicinal products (cannabis oil and matched placebo) used in the trial.

Open access publishing was facilitated by Southern Cross University, as part of the Wiley‐Southern Cross University agreement via the Council of Australasian University Librarians.

## Disclosure

All authors have approved the final version.

## Ethics Statement

The trial was approved by the Human Research Ethics Committees of Southern Cross University (approval number 2022/146) and Griffith University (approval number 2023/137).

## Consent

All participants were adults (≥ 18 years) and provided written informed consent prior to enrolment. Parent/guardian consent was not applicable to this study. The consent process conformed to the approved Human Research Ethics Committee protocols and was documented in participant study files.

## Conflicts of Interest

Inna Kurlyandchik received a PhD scholarship funded equally by Southern Cross University (SCU) and Little Green Pharma Ltd (LGP). LGP supplied the investigational medicinal products (cannabis oil and matched placebo) for the trial but had no role in study design, conduct, data collection, data management, statistical analysis, interpretation of results, manuscript preparation, review, approval or the decision to submit for publication. Janet Schloss provided research funds to support trial conduct but was not involved in study management, data analysis or interpretation. All other authors declare no conflicts of interest.

## Supporting Information

Additional supporting information can be found online in the Supporting Information section.

## Supporting information


**Supporting Information** Supporting Information SI1: Complete list of inclusion and exclusion criteria for participant eligibility. Supporting Information SI2: Detailed titration schedule showing dose escalation protocol over the 4‐week titration period. Supporting Information SI3: Trial layout diagram and complete assessment schedule showing timing of all study visits and outcome measures. Supporting Information SI4: Average weekly doses for treatment and placebo groups during the titration phase.

## Data Availability

The data that support the findings of this study are available from Southern Cross University. Restrictions apply to the availability of these data, which were used under licence for this study. Data are available from the corresponding author with the permission of Southern Cross University.
