# Supplementary material for: Feasibility, Safety and Preliminary Efficacy of 1:1 THC:CBD Cannabis Oil for Fibromyalgia Symptoms: Results From a Randomised, Double‐Blind, Placebo‐Controlled Pilot Trial
Source: Pain Res Manag. 2026 May 16;2026:7311235. doi: 10.1155/prm/7311235 (PMC13179683; doi:10.1155/prm/7311235)

**Supplementary Material SM1. Inclusion and Exclusion Criteria**

# ELIGIBILITY CRITERIA

## Inclusion criteria

1. Age ≥ 18 years
2. Able to give written informed consent
3. Able to complete subject-reported questionnaires per the investigator's judgement
4. The subject does not have a disorder that would otherwise explain the pain
5. At screening, subjects must meet the 2016 American College of Rheumatology (ACR) criteria for FMS:
    - Widespread pain index (WPI) ≥ 7 and symptom severity score (SSS) ≥ 5, or WPI 4-6 and SSS ≥ 9
    - Generalised pain, defined as pain in at least 4 of 5 regions, is present.
    - Symptoms have been generally present for at least 3 months
6. Average daily pain score (ADPS) ≥ 4 on the 11-point numeric rating scale (NRS) over the past seven days before randomisation (based on completion of at least four daily pain diaries during the 7-day baseline period before randomisation)
7. Participants are willing to ensure that they or their partner use a highly effective contraception method[1] (or combination of two less effective methods[2]) during the study and for 4 weeks thereafter (applicable to heterosexual female patients of childbearing potential[3] and fertile male patients[4] whose partners are of childbearing potential).

## Exclusion criteria

*Pre-existing conditions:*

1. Clinically significant unstable neurologic, psychiatric, ophthalmologic, hepatobiliary, respiratory, hematologic illness or cardiovascular disease (e.g., severe hypotension, uncontrolled cardiac

---

[1] Highly effective birth control methods include:
- combined (estrogen and progestogen containing) hormonal contraception associated with inhibition of ovulation: oral, intravaginal, transdermal
- progestogen-only hormonal contraception associated with inhibition of ovulation: oral, injectable, implantable
- intrauterine device (IUD)
- intrauterine hormone-releasing system (IUS)
- bilateral tubal occlusion
- vasectomised partner (provided that vasectomised partner is the sole sexual partner of the trial participant)
- sexual abstinence (refraining from heterosexual intercourse during the entire period of risk associated with the study treatments).

[2] Less effective birth control methods include:
- progestogen-only oral hormonal contraception, where inhibition of ovulation is not the primary mode of action
- male or female condom with or without spermicide
- cap, diaphragm, or sponge with spermicide

[3] For the purpose of this trial, a woman is considered of childbearing potential, i.e., fertile, following menarche and until becoming post-menopausal unless permanently sterile. Permanent sterilisation methods include hysterectomy, bilateral salpingectomy, and bilateral oophorectomy. A postmenopausal state is defined as no menses for 12 months without an alternative medical cause.

[4] For the purpose of this trial, a man is considered fertile after puberty unless permanently sterile by bilateral orchidectomy.

arrhythmia, or myocardial infarction) or any other concurrent disease within 12 months before screening that in the opinion of the investigator would interfere with study participation or assessment of safety and tolerability.

2. Subjects who are at risk of suicide as defined by their responses to the Columbia-Suicide Severity Rating Scale (C-SSRS) or the investigator's opinion. *Note: Patients answering "yes" to any of the questions about active suicidal ideation/intent/behaviours occurring within the past 12 months will be excluded (C-SSRS Suicide Ideation section – Questions 3, 4, or 5; C-SSRS Suicidal Behaviour section, any of the suicide behaviours questions). Such patients will be directed to a mental health support service.*
3. Current moderate severe to severe depression or anxiety disorders as assessed by the Depression Patient Health Questionnaire-9 (PHQ-9, total score of >10) and Generalized Anxiety Disorder-7 questionnaire (GAD-7, total score of >10). Still, mild to moderate depression (PHD-9 total score of 0-10) or anxiety disorders (GAD-7 total score of 0-10) are permitted provided that the investigator assesses the patient as clinically stable and appropriate for entry into the study.
4. Any diagnosis of lifetime psychotic or bipolar disorder.
5. Subjects with pain due to other conditions (e.g., diabetic peripheral neuropathic pain or post-herpetic neuralgia) that, in the investigator's opinion, would confound assessment or self-evaluation of the pain associated with FMS.
6. Subjects with pain due to any widespread inflammatory musculoskeletal disorder (e.g., rheumatoid arthritis, lupus) or widespread rheumatic disease other than FMS.
7. Known hypersensitivity medical cannabis products. *Note: Prior exposure is allowed, as long as hypersensitivity to cannabis was not observed.*
8. Known tree nut allergy, particularly walnut allergy.
9. Abnormal investigative tests and laboratory values judged by the investigator to be clinically significant at screening, with particular focus on:
   a. Abnormal renal function defined as calculated creatinine clearance (CrCl) < 60 mL/min determined by the central laboratory using the modified Cockcroft-Gault equation; blood urea nitrogen> 1.5 × upper limit of normal (ULN); creatine kinase > 3.0 × ULN; serum creatinine > 1.6 mg/dL (> 141.4 µmol/L).
   b. Abnormal liver function defined as aspartate aminotransferase (AST) > 2.0 × ULN, alanine aminotransferase (ALT) > 2.0 × ULN; alkaline phosphatase > 1.5 × ULN; total bilirubin> 1.2 × ULN. If a subject has total bilirubin > 1.2 ULN, unconjugated and conjugated bilirubin fractions should be analysed, and only subjects documented to have Gilbert's syndrome may be enrolled.
10. Any history of a malignant neoplasm other than benign skin cancers within the past five years.
11. Pregnant or breastfeeding, or intend to become pregnant during the study period or refusing to do pregnancy tests through the study.

*Medications:*

12. Current antipsychotic use (except for low-dose antipsychotics prescribed by a physician to treat sleep disorders).
13. Current chemotherapy, radiation, immune suppressant therapy, or immunotherapy.
14. Current warfarin administration.
15. Unable to undergo pre-study washout (30 days or less as assessed by urinary THC test) of prohibited concomitant medications/substances (including cannabis/medicinal cannabis products).
16. Subject is currently enrolled in or has not yet completed at least 30 days since ending another investigational device or drug study or is receiving other investigational agents.

Southern Cross University
National Centre for Naturopathic Medicine

Supplementary Material SM2. Sample Titration Schedule

**Please note: You can stay at any particular dose for more than one day before deciding if you want to increase or decrease your dose. Only increase or decrease your dose by 0.25 ml at a time.**

| Days | Daily total bedtime dose | How do I feel? | | What to do? |
|---|---|---|---|---|
| 1-2 | 0.25 ml | I noticed significant side effects | → | Contact the researchers |
| | | I noticed improvements of my symptoms, with no significant side effects | → | Stay at this dose – this is your dose of tolerance! |
| | | I did not notice improvements of my symptoms, or any significant side effects | → | Increase your bedtime dose by 0.25ml |
| 3-4 | 0.5 ml | I noticed significant side effects | → | Contact the researchers |
| | | I noticed improvements of my symptoms, with no significant side effects | → | Stay at this dose – this is your dose of tolerance! |
| | | I did not notice improvements of my symptoms, or any significant side effects | → | Increase your bedtime dose by 0.25ml |
| 5-6 | 0.75 ml | I noticed significant side effects | → | Contact the researchers |
| | | I noticed improvements of my symptoms, with no significant side effects | → | Stay at this dose – this is your dose of tolerance! |
| | | I did not notice improvements of my symptoms, or any significant side effects | → | Increase your bedtime dose by 0.25ml |
| 7-8 | 1 ml | I noticed significant side effects | → | Contact the researchers |
| | | I noticed improvements of my symptoms, with no significant side effects | → | Stay at this dose – this is your dose of tolerance! |
| | | I did not notice improvements of my symptoms, or any significant side effects | → | Increase your bedtime dose by 0.25ml |
| 9-10 | 1.25 ml | I noticed significant side effects | → | Contact the researchers |
| | | I noticed improvements of my symptoms, with no significant side effects | → | Stay at this dose – this is your dose of tolerance! |
| | | I did not notice improvements of my symptoms, or any significant side effects | → | Increase your bedtime dose by 0.25ml |
| 11-12 | 1.5 ml | I noticed significant side effects | → | Contact the researchers |
| | | I noticed improvements of my symptoms, with no significant side effects | → | Stay at this dose – this is your dose of tolerance! |
| | | I did not notice improvements of my symptoms, or any significant side effects | → | Increase your bedtime dose by 0.25ml |
| 13-14 | 1.75 ml | I noticed significant side effects | → | Contact the researchers |
| | | I noticed improvements of my symptoms, with no significant side effects | → | Stay at this dose – this is your dose of tolerance! |
| | | I did not notice improvements of my symptoms, or any significant side effects | → | Increase your bedtime dose by 0.25ml |

Southern Cross University
National Centre for Naturopathic Medicine

**Please note: You can stay at any particular dose for more than one day before deciding if you want to increase or decrease your dose. Only increase or decrease your dose by 0.25 ml at a time.**

| Days | Daily total bedtime dose | How do I feel? | | What to do? |
|---|---|---|---|---|
| 15-16 | 2 ml | I noticed significant side effects | → | Contact the researchers |
| | | I noticed improvements of my symptoms, with no significant side effects | → | Stay at this dose – this is your dose of tolerance! |
| | | I did not notice improvements of my symptoms, or any significant side effects | → | Increase your bedtime dose by 0.25ml |
| 17-18 | 2.25 ml | I noticed significant side effects | → | Contact the researchers |
| | | I noticed improvements of my symptoms, with no significant side effects | → | Stay at this dose – this is your dose of tolerance! |
| | | I did not notice improvements of my symptoms, or any significant side effects | → | Increase your bedtime dose by 0.25ml |
| 19-20 | 2.5 ml | I noticed significant side effects | → | Contact the researchers |
| | | I noticed improvements of my symptoms, with no significant side effects | → | Stay at this dose – this is your dose of tolerance! |
| | | I did not notice improvements of my symptoms, or any significant side effects | → | Increase your bedtime dose by 0.25ml |
| 21-22 | 2.75 ml | I noticed significant side effects | → | Contact the researchers |
| | | I noticed improvements of my symptoms, with no significant side effects | → | Stay at this dose – this is your dose of tolerance! |
| | | I did not notice improvements of my symptoms, or any significant side effects | → | Increase your bedtime dose by 0.25ml |
| 23-24 | 3 ml | I noticed significant side effects | → | Contact the researchers |
| | | I noticed improvements of my symptoms, with no significant side effects | → | Stay at this dose – this is your dose of tolerance! |
| | | I did not notice improvements of my symptoms, or any significant side effects | → | Increase your bedtime dose by 0.25ml |
| 25-26 | 3.25 ml | I noticed significant side effects | → | Contact the researchers |
| | | I noticed improvements of my symptoms, with no significant side effects | → | Stay at this dose – this is your dose of tolerance! |
| | | I did not notice improvements of my symptoms, or any significant side effects | → | Increase your bedtime dose by 0.25ml |
| 27-28 | 3.5 ml | I noticed significant side effects | → | Contact the researchers |
| | | I noticed improvements of my symptoms, with no significant side effects | → | Stay at this dose – this is your dose of tolerance! |
| | | I did not notice improvements of my symptoms, or any significant side effects | → | You have reached the maximum dose. Stay at this dose for the rest of the trial. |

## Supplementary Material SM3. Trial Layout and Assessment Schedule

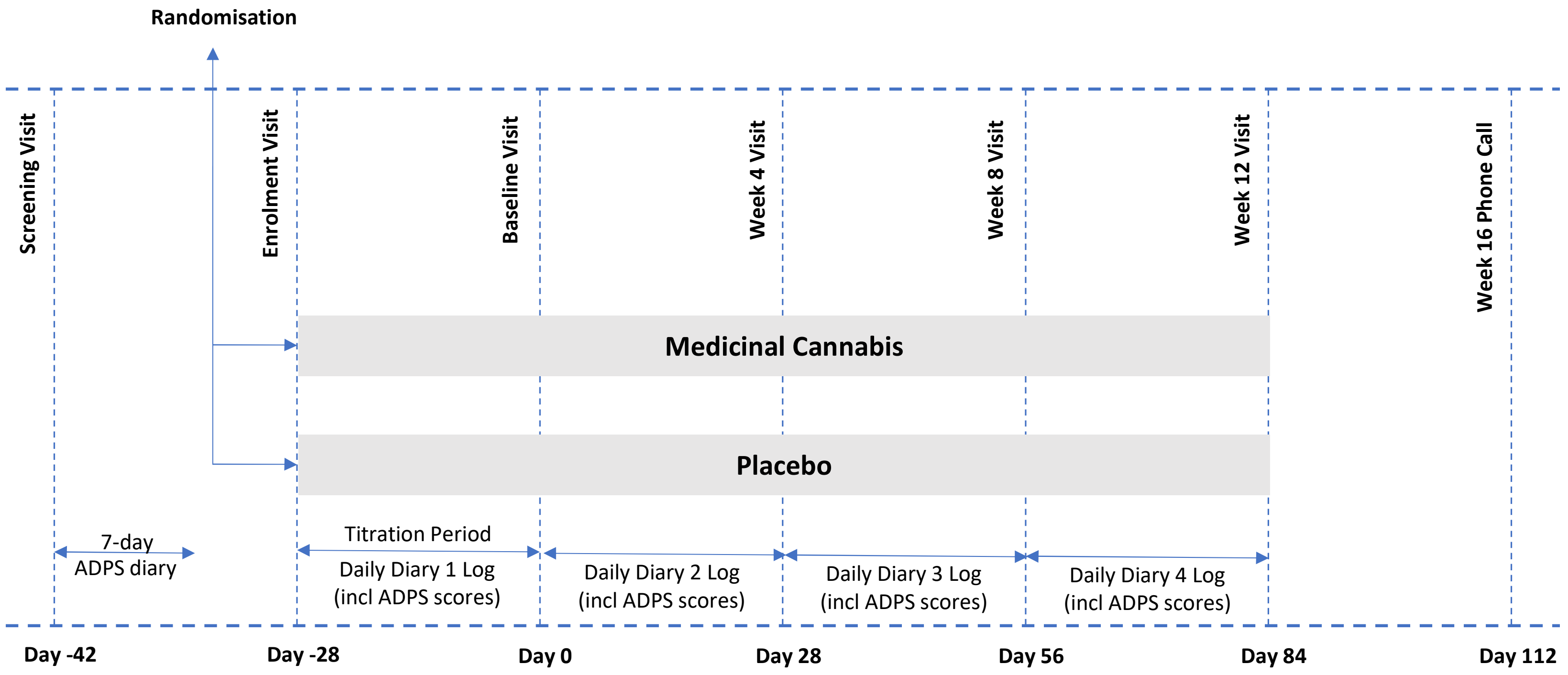


**Day -42**

- Consent
- Confirmation of FMS diagnosis (ACR)
- PHQ-9, GAD-7, C-SSRS
- Hight, Weight, BP, HP
- Medication check
- Blood sample (FBC, E/LFT)
- Pregnancy test
- DOA test
- 7-day ADPS diary provided

**Day -28**

- Fasting blood sample
- Weight, BP, HP
- Medication check
- Pregnancy test
- DOA test
- Questionnaires completed (FIQ, SF-36, HADS, PSQI, MFI-20, C-SSRS)
- Diary 1 provided
- IMP dispensed

**Day 0**

- Fasting blood sample
- Weight, BP, HP
- Medication check
- Pregnancy test
- DOA test
- Questionnaires completed (FIQ, SF-36, HADS, PSQI, MFI-20, C-SSRS)
- Diary 2 provided
- IMP returned for counting
- IMP dispensed

**Day 28**

- Fasting blood sample
- Weight, BP, HP
- Medication check
- Pregnancy test
- DOA test
- Questionnaires completed (FIQ, SF-36, HADS, PSQI, MFI-20, C-SSRS)
- Diary 3 provided
- IMP returned for counting
- IMP dispensed

**Day 56**

- Fasting blood sample
- Weight, BP, HP
- Medication check
- Pregnancy test
- DOA test
- Questionnaires completed (FIQ, SF-36, HADS, PSQI, MFI-20, C-SSRS)
- Diary 4 provided
- IMP returned for counting
- IMP dispensed

**Day 84**

- Fasting blood sample
- Weight, BP, HP
- Medication check
- Pregnancy test
- DOA test
- Questionnaires completed (FIQ, SF-36, ADPS, HADS, PSQI, MFI-20, C-SSRS)
- IMP returned for counting
- End of Trial Evaluation

**Day 112**

- Final follow-up call

**Supplementary Material SM4. Average weekly doses during titration by group**

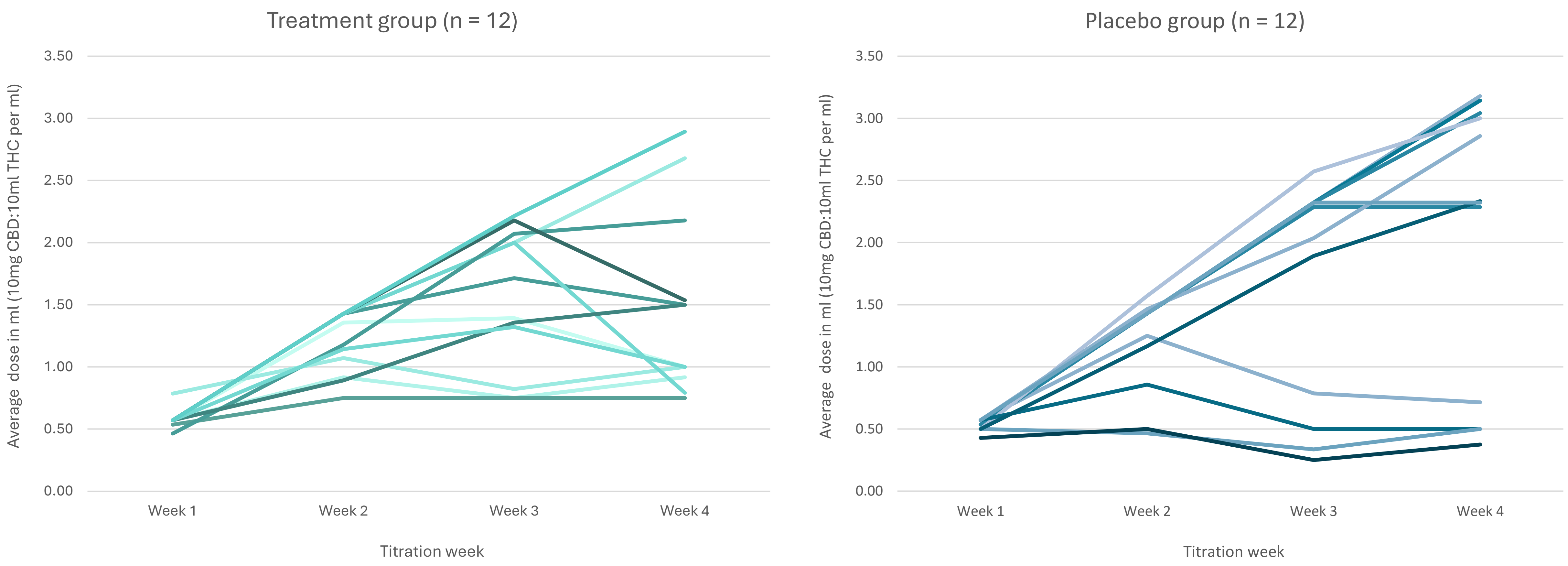

Supplement: Supplementary file 1 — Supporting Information Supporting Information SI1: Complete list of inclusion and exclusion criteria for participant eligibility. Supporting Information SI2: Detailed titration schedule showing dose escalation protocol over the 4‐week titration period. Supporting Information SI3: Trial layout diagram and complete assessment schedule showing timing of all study visits and outcome measures. Supporting Information SI4: Average weekly doses for treatment and placebo groups during the titration phase. [file PRM-2026-7311235-s001.pdf]
